# Skin Globotriaosylceramide 3 Load Is Increased in Men with Advanced Fabry Disease

**DOI:** 10.1371/journal.pone.0166484

**Published:** 2016-11-16

**Authors:** Nurcan Üçeyler, Nils Schröter, Waldemar Kafke, Daniela Kramer, Christoph Wanner, Frank Weidemann, Claudia Sommer

**Affiliations:** 1 Department of Neurology, University of Würzburg, Würzburg, Germany; 2 Department of Internal Medicine I, University of Würzburg, Würzburg, Germany; 3 Würzburg Fabry Center for Interdisciplinary Therapy (FAZIT), University of Würzburg, Würzburg, Germany; Weizmann Institute of Science, ISRAEL

## Abstract

**Background:**

The X-chromosomally linked life-limiting Fabry disease (FD) is associated with deposits of the sphingolipid globotriaosylceramide 3 (Gb3) in various tissues. Skin is easily accessible and may be used as an additional diagnostic and follow-up medium. Our aims were to visualize skin Gb3 deposits in FD patients applying immunofluorescence and to determine if cutaneous Gb3 load correlates with disease severity.

**Methods:**

At our Fabry Center for Interdisciplinary Therapy we enrolled 84 patients with FD and 27 healthy controls. All subjects underwent 5-mm skin punch biopsy at the lateral lower leg and the back. Skin samples were processed for immunohistochemistry using antibodies against CD77 (i.e. Gb3). Cutaneous Gb3 deposition was quantified in a blinded manner and correlated to clinical data.

**Results:**

We found that Gb3 load was higher in distal skin of male FD patients compared to healthy controls (p<0.05). Men (p<0.01) and women (p<0.05) with a classic FD phenotype had higher distal skin Gb3 load than healthy controls. Men with advanced disease as reflected by impaired renal function, and men and women with small fiber neuropathy had more Gb3 deposits in distal skin samples than males with normal renal function (p<0.05) and without small fiber neuropathy. Gb3 deposits were not different between patients with and without enzyme replacement therapy.

**Conclusions:**

Immunofluorescence on minimally invasive skin punch biopsies may be useful as a tool for assessment and follow-up in FD patients.

## Background

The lysosomal storage disorder Fabry disease (FD) is an X-linked recessive hereditary disease caused by mutations in the gene encoding the enzyme α-galactosidase A (α-GAL). These mutations lead to a reduction or loss of α-GAL activity, which results in the accumulation of sphingolipids, particularly globotriaosylceramide-3 (Gb3), in kidneys, heart, and the nervous system [1].

In skin, Gb3 may accumulate in vascular endothelium, smooth muscles, fibroblasts, and eccrine sweat glands [2–8] and it has been localized to lysosomes using electron microscopy [9]. In a phase 3 clinical trial on enzyme replacement therapy (ERT), clearance of Gb3 was shown from vascular endothelium and to a lesser degree from dermal smooth muscle cells and perineurium [10].

So far only few studies investigating Gb3 load in skin of FD patients have been published, and in these studies electron microscopy was used [9]. This technique, however, is costly and time-consuming, and only a very small section of the affected skin can be examined. While in several studies light microscopy was successfully applied to investigate Gb3 load in kidneys [11,12], no such investigations were reported in skin of FD patients except for one study using semithin sections and showing deposits assumed to be Gb3 [10]. We set out to use fluorescence microscopy to quantify the Gb3 load in skin punch biopsies of patients with FD, and assessed its association with disease severity and other clinical parameters, including ERT.

## Patients and Methods

### Subjects

Our study was approved by the Würzburg Medical Faculty Ethics Committee. All study participants gave written informed consent before enrollment. We investigated 84 consecutive FD patients who reported at the Würzburg Fabry Center for Interdisciplinary Therapy (FAZIT) between 2007 and 2013. FAZIT is a tertiary referral center, where patients are seen from all over Germany mainly to confirm the diagnosis, decide on treatment, and for follow-up. Adult patients ≥18 years were included if FD was confirmed by α-GAL assay and a genetic test. The patient group consisted of 38 men (median age 41 years, range 18–71 years) and 46 women (median age 41 years, range 20–70 years). Data on the peripheral nervous system [13] and on cerebrovascular findings [14,15] of some of these patients have been described elsewhere. Additionally, we recruited 27 healthy volunteers as controls. This control group consisted of 14 men (median age 54 years, range 23–76 years) and 13 women (median age 57, range 20–81 years).

### Clinical examination and laboratory tests

As part of the routine work-up all patients underwent neurological examination and were assessed with a standardized pain questionnaire (Neuropathic Pain Symptom Inventory, NPSI [16,17]). After exclusion of large fiber neuropathy by clinical examination and routine neurophysiological studies, the presence of a small fiber neuropathy (SFN) was determined according to published criteria [18] using standard quantitative sensory testing at the right dorsal foot [19] and skin punch biopsy. Kidney function was assessed by the glomerular filtration rate (GFR). Normal renal function was defined as GFR ≥60 ml/min/1.73 m^2^ [20–22]. Cardiomyopathy was diagnosed if typical findings like cardiac fibrosis and left ventricular hypertrophy were present.

### Skin punch biopsies

From all study participants a 5-mm skin punch biopsy (apparatus by Stiefel GmbH, Offenbach, Germany) was obtained under sterile conditions and after topical anesthesia with 1% scandicaine [23]. Biopsies were taken from the lateral lower leg 10 cm proximal of the ankle and from the back at Th10 level. These sites were chosen to keep consistency with our previous studies, where we assessed length-dependency of the small nerve fiber reduction. Skin samples were processed as described earlier [24].

### Intraepidermal nerve fiber density (IENFD)

For IENFD quantification skin specimens were fixed in fresh 4% buffered paraformaldehyde (pH 7.4) for 30 minutes, washed in phosphate buffer, and stored in 10% sucrose with 0.1M phosphate buffer. Skin samples were then embedded in Tissue Tek^®^, frozen in 2-methylbutane cooled in liquid nitrogen and stored at -80°C before further processing. Forty-μm (for IENFD count) and 10-μm (for CD77 and double stains) cryostat cryostat sections were immunoreacted with antibodies against the pan-axonal marker protein-gene product 9.5 (PGP 9.5; Ultraclone, UK, 1:800) with goat anti-rabbit IgG labelled with cyanine 3.18 fluorescent probe to visualize nerve fibers. Blood vessels were identified by a monoclonal antibody to factor VIII (von Willebrand factor, Dako, USA, 1:200). Three biopsy sections per site were analyzed with a Zeiss Axiophot 2 microscope (Axiophot2, Zeiss, Germany) with a CCD camera (Visitron Systems, Tuchheim, Germany) and SPOT advanced software (Windows Version 4.5, Diagnostic Instruments, Inc, Sterling Heights, USA) on coded slides by an observer blinded to the identity of the specimen. Epidermal nerve fibers were counted following published rules [25].

### Quantification of Gb3 immunoreactivity

**Fig 1:**
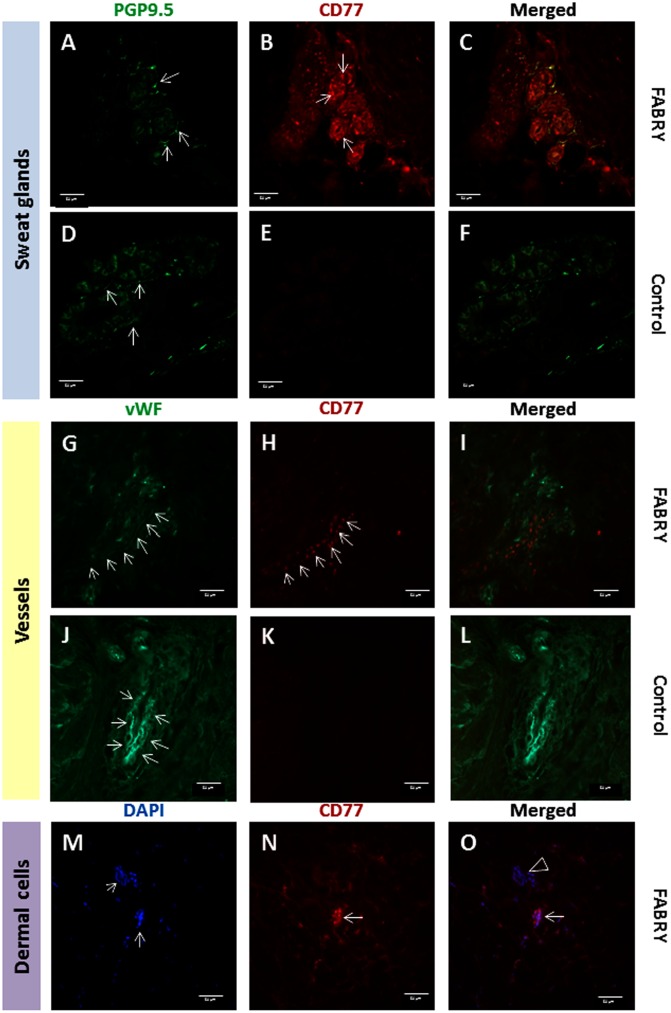
Representative photomicrograph of skin punch biopsy samples from the lower leg of patients with Fabry disease and healthy controls are shown using double immunofluorescence stains. Globotriaosylceramide 3 (Gb3) deposition is shown in cutaneous sweat glands, blood vessels, and dermal cells. Ten-μm skin sections were immunoreacted with an antibody against the pan-axonal marker protein-gene product 9.5 (PGP 9.5; Ultraclone, UK, 1:800) to visualize nerve fibers; with an antibody agains factor VIII (von Willebrand factor, vWF; Dako, USA, 1:200) to show endothelium of blood vessels; with an antibody against the CD77 antigen (i.e. Gb3; BD Pharmingen, Heidelberg, Germany, 1:100) to visualize Gb3 deposits. Nuclei were visualized using 4,6-diamidin-2-phenylindol (DAPI; Boehringer, Mannheim). Dermal sweat glands of a Fabry patient (A) and a healthy control (D) are densely innervated (arrows pointing at PGP9.5 immunoreactive nerve fibers). In the sweat gland tubules of the Fabry patient dense Gb3 deposits are seen (arrows) while no such deposits are found in the healthy control (E). C) and F) show the respective merged photomicrographs. Dermal vessels of a Fabry patient (G) and a healthy control (J) are determined by the endothelial vWF (arrows). The vessel wall of the Fabry patient contains Gb3 deposits (H; arrows), while no such deposits are seen in the vessel walls of the healthy control (K). I) and L) show the respective merged photomicrographs. Dermal cells are determined by nuclear staining in skin of a Fabry patient (M, arrows). Intracellular Gb3 deposits are found in some (N, arrow) but not all (O, arrow head) dermal cells. Bar = 50 μm.

For quantification of the Gb3 load 10-μm cryostat sections were immunoreacted with antibodies against the CD77 antigen (i.e. Gb3 [26]) (BD Pharmingen cat# 551352, Heidelberg, Germany, 1:100). Antigen retrieval was not necessary. For each immunoreaction a negative control without the primary antibody was used and assessments were performed only if the negative control did not show specific staining. Three photomicrographs per case including the entire dermis of each of the three sections per subject were captured using a 20x objective and were calibrated (2724 pixels/μm; Fig 1). Photomicrographs were assessed off-line semiautomatically using Image J software (Version 1.45; http://imagej.nih.gov/ij/download.html). The dermis on each photomicrograph was covered with three quadrangles as “regions of interest” (ROI) with a total of 4.194.304 pixels (with 7407 pixels/20 μm). The area covered by Gb3 was determined as % of this entire ROI.

### Statistical analysis

IBM SPSS Statistics 23.0 software (Ehningen, Germany) was used for statistical analysis; Graph Pad Prism 3.0 (GraphPad Software, Inc., San Diego, USA) was applied for graphical illustrations. Since data were not normally distributed, the non-parametric Mann-Whitney-U-test was applied for group comparisons. Results are illustrated as scatter plots giving individual data. For sensitivity and specificity analyses, receiver operating characteristic (ROC) curves were calculated. Additionally, linear regression analysis was performed. P<0.05 was considered significant.

## Results

### Demographic findings and skin innervation

Table 1 gives baseline data of the patient population; S1 Table shows individual general data and S2 Table individual kidney data. The patient cohort consisted of n = 77 patients with classical and n = 7 non-classical phenotype (n = 5 male, n = 2 female) [27]. There was no intergroup difference as for age between FD patients and controls (p>0.05). Thirty patients (19 men, 11 women) were on ERT. The median time on ERT was 3.1 years (0.2–8.9) and 21 of these patients were on agalsidase-beta. Median IENFD was reduced in distal skin of men (0.5 fibers/mm, 0–3) and women with FD (3.7 fibers/mm, 1.8–4.5) compared to our laboratory normative values (lower leg 9+/-3 fibers/mm; Table 1).

**Table 1:** Characteristics of patient population.

	Fabry M	Fabry F
Number of patients	38	46
Median age (range) years	40 (18–71)	41 (20–70)
Median time since diagnosis years (range)	3 (0–9)	4 (0–7)
Number of patients with Fabry-associated pain	22/38 (58%)	27/46 (59%)
Number of patients with hypo-/anhidrosis	22/38 (58%)	15/46 (33%)
Number of patients with SFN	21/38 (55%)	16/46 (35%)
Number of patients with cardiomyopathy	19/38 (50%)	12/46 (26%)
Median NPSI sum score (range)	0.2 (0–0.6)	0.1 (0–0.5)
Median ADS score (range)	17 (0–40)	10 (0–45)
Number of patients on ERT	19/38 (50%)	11 (24%)
Median time since ERT (range)	3 (0.1–9)	4 (0.2–7)
Median IENFD lower leg (fibers/mm; range)*	0.5 (0–3)	3.7 (1.8–4.5)
Median IENFD back (fibers/mm; range)*	16.3 (0.6–30.6)	23.8 (0.8–53.8)

*Laboratory normative values: Lower leg: 9+/-3 fibers/mm; back: 12 +/- 4 fibers/mm.

### Distal skin Gb3 load is higher in men with FD compared to women and to controls

**Fig 2:**
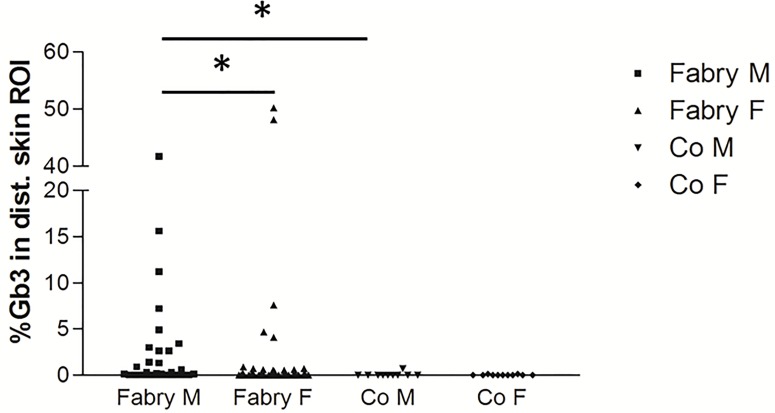
Scatter plots illustrate quantification of Gb3 load in CD77 stains of skin punch biopsy specimens from the lower leg of patients with Fabry disease (FD) compared to controls. Skin Gb3 load is higher in male FD patients than in female patients and male healthy controls. Similarly, some female FD patients have a higher skin Gb3 load than female controls, while group medians are not different. Number of subjects scatter plot from left to right: n = 36, n = 43, n = 10, n = 11. Abbreviations: Co: Controls; F: Female; Gb3 = globotriaosylceramide 3; M: Male; ROI: investigated region of interest on skin punch biopsy specimens. *p<0.05.

**Fig 3:**
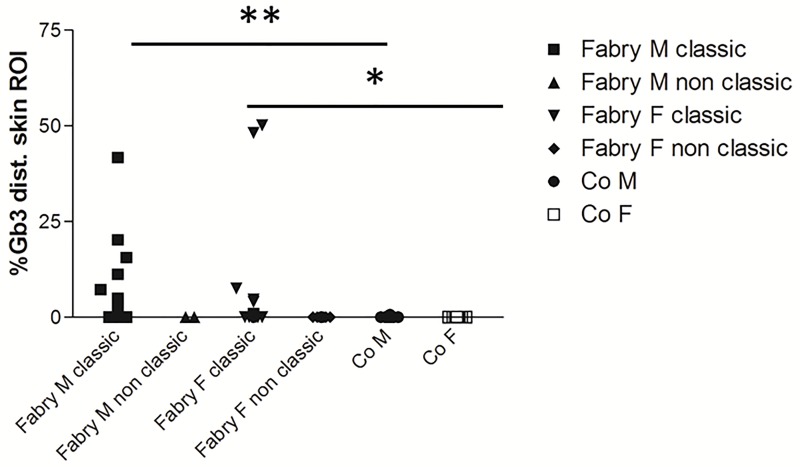
Scatter plots illustrate quantification of Gb3 load in CD77 stains of skin punch biopsy specimens from the lower leg of patients with Fabry disease (FD) of classical and non-classical phenotype stratified for gender. Skin Gb3 load is higher in male and female FD patients with classical phenotype compared to respective healthy controls. Patients with non-classical phenotype do not differ from controls and only one patient out of seven exceeded the threshold of >0.05% ROI Gb3 deposition in distal skin. Number of subjects from left to right: n = 32, n = 2, n = 5, n = 39, n = 5, n = 10, n = 11. Abbreviations: Co = controls; F = female; Gb3 = globotriaosylceramide 3; M = male; ROI: investigated region of interest on skin punch biopsy specimens; SFN = small fiber neuropathy. *p<0.05, **p<0.01.

Gb3 deposits were easily detectable in sweat gland tubules, blood vessel walls, and dermal cells of the lower leg skin of FD patients, but not in healthy controls (Fig 1). In skin samples from the back, only 26 FD patients (n = 11 men, n = 15 women) showed Gb3 deposits >0.05% ROI, such that we chose the lower leg specimen for further analyses. Using this threshold 20/38 (53%) men and 19/46 (41%) women with FD were positive for Gb3 in distal skin, while in controls only n = 2 male and female subjects each exceeded this threshold value. In lower leg skin male FD patients (n = 38) had more Gb3 deposits than female FD patients (n = 46; p<0.05) and male healthy controls (p<0.05; Fig 2). The median Gb3 load in distal skin of all female FD patients did not differ from female healthy controls (Fig 2). However, considering patients with classic phenotype only, men and women had a higher Gb3 load in distal skin compared to controls (male: p<0.01; female: p<0.05; Fig 3). Deposits were also more pronounced compared to patients with non-classic phenotype, however, with the low number of patients in this subgroup (n = 7) significance was not reached. Among these only one female patient had a distal skin Gb3 deposition of >0.05% ROI.

### Skin Gb3 load increases with disease severity and distinguishes male patients from controls with high specificity

**Fig 4:**
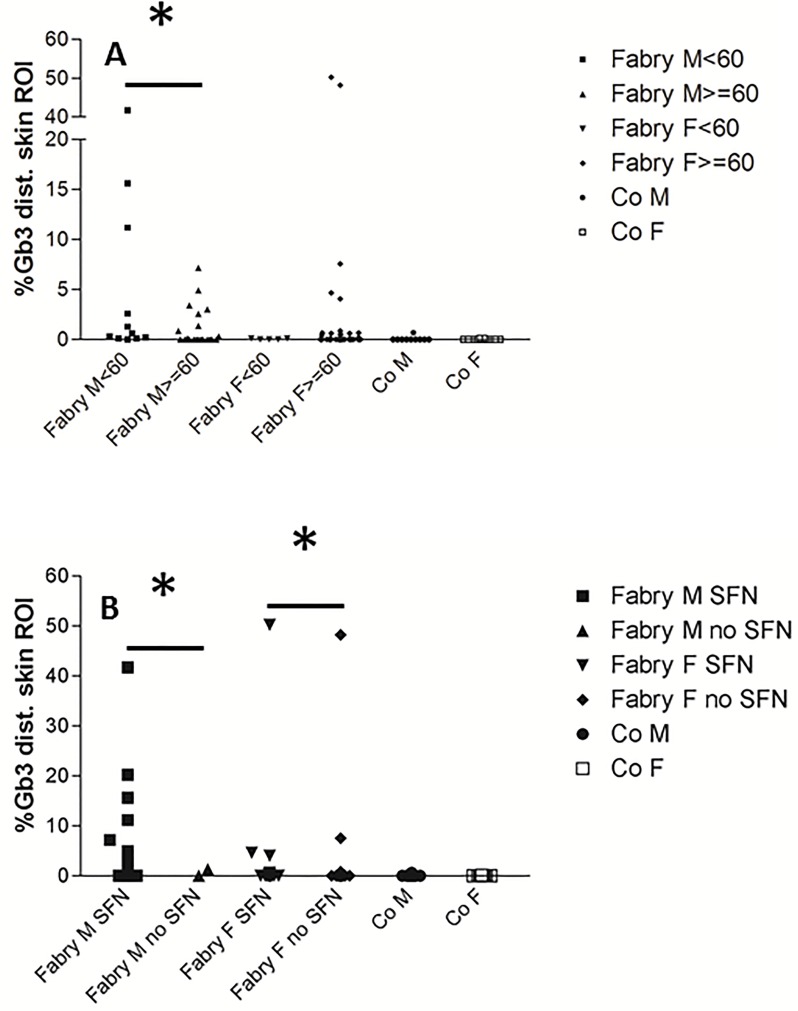
A) Scatter plots illustrate quantification of Gb3 load in CD77 stains of skin punch biopsy specimens from the lower leg of patients with Fabry disease (FD) and impaired versus normal renal function as measured by the glomerular filtration rate (≥60 ml/min/1.73 m^2^: normal; <60 ml/min/1.73 m^2^: impaired). Skin Gb3 load is higher in male FD patients with impaired renal function than in men with normal renal function. Number of subjects from left to right: n = 12, n = 22, n = 5, n = 39, n = 10, n = 11. B) Scatter plots show the quantification of Gb3 load in CD77 stains of skin punch biopsy specimens from the lower leg of patients with FD stratified for the presence of small fiber neuropathy (SFN) and compared to healthy controls. Male and female FD patients with SFN have more dermal Gb3 deposits in distal skin than patients without SFN. Number of subjects per box from left to right: n = 34, n = 2, n = 19, n = 24, n = 10, n = 11. Abbreviations: F = female; Gb3 = globotriaosylceramide 3; M = male; ROI: investigated region of interest on skin punch biopsy specimens; SFN = small fiber neuropathy. *p<0.05.

**Fig 5:**
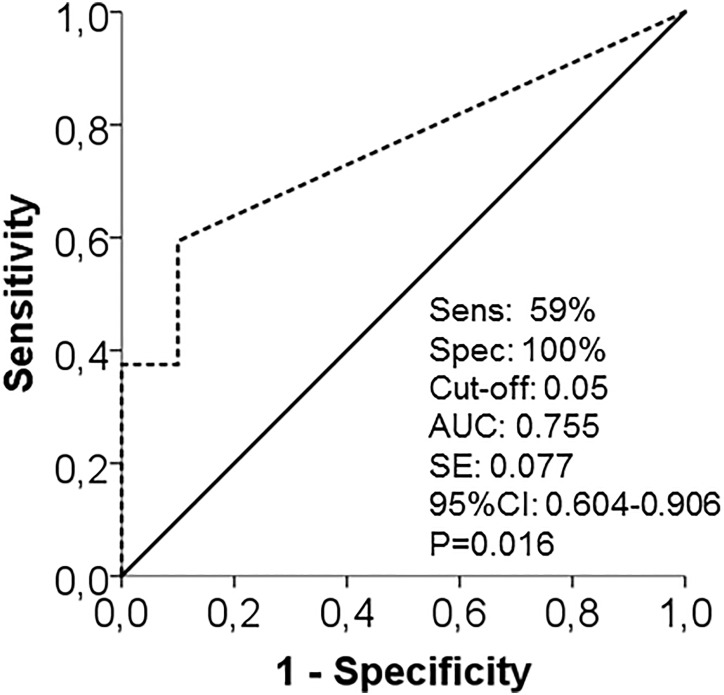
Receiver operating characteristic (ROC) curve is shown for distal skin Gb3 load in male FD patients compared to healthy controls. The relative frequency of ‘‘true positive” values on the y-axis (i.e., sensitivity) is plotted against the relative frequency of ‘‘true negative” values on the x-axis (i.e., 1-sensitivity). Note that the highest sensitivity (59%) and specificity (100%) for FD was found with the cut-off value set at 0.05. Abbreviations: AUC = area under the curve; CI = confidence interval; F = female; Gb3 = globotriaosylceramide 3; M = male; ROI: investigated region of interest on skin punch biopsy specimens; SE = standard error.

**Fig 6:**
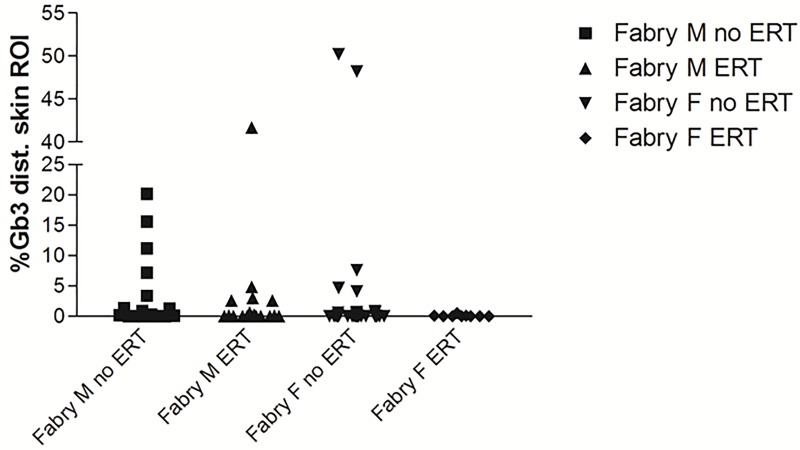
Scatter plots show the quantification of Gb3 load in CD77 stains of skin punch biopsy specimens from the lower leg of patients with Fabry disease (FD) stratified for enzyme replacement therapy (ERT). Male and female FD patients without ERT did not differ from patients on ERT. Number of subjects from left to right: n = 16; n = 17, n = 18, n = 35). Abbreviations: ERT = enzyme replacement therapy; F = female; Gb3 = globotriaosylceramide 3; M = male; n.s. = not significant; ROI: investigated region of interest on skin punch biopsy specimens. *p<0.05.

When stratifying our FD patient group for renal function, men with impaired kidney function (n = 12) had more Gb3 deposits in distal skin than men with normal renal function (n = 26, p<0.05; Fig 4A). With only n = 5 women with impaired renal function, the sample size was too small to detect a significant difference for female FD patients (Fig 4A). Also, male (n = 36) and female (n = 20) FD patients with SFN had more Gb3 deposits in distal skin than respective patients without SFN (p<0.05 each; Fig 4B). ROC analysis revealed that with a cut-off at 0.05 (AUC: 0.755; SE: 0.077; 95% CI: 0.604–0.906; p<0.05; Fig 5), dermal Gb3 load in lower leg skin biopsies reached a sensitivity of only 59%, but a specificity of 100% for the detection of FD when regarding the entire male patient group. Linear regression analysis investigating a potential relationship between distal skin Gb3 load and age, phenotype, GFR, cardiomyopathy, and IENFD did not reveal relevant correlations (data not shown). Male (n = 19) and female (n = 35) FD patients without ERT did not differ statistically in Gb3 load of distal skin when compared with patients on ERT (Fig 6). While this may be due to the low number of cases in the subgroups, it is interesting that none of the women on ERT showed Gb3 deposits compared to five in the untreated group with a similar trend in the male patients (Fig 6).

## Discussion

In this single-center study we show that Gb3 deposits can be visualized in human skin punch biopsies by immunofluorescence, that dermal Gb3 deposits indicate FD with high specificity, and that the skin Gb3 load is particularly high in male FD patients with advanced disease and classic phenotype.

There is only limited experience with immunofluorescence for Gb3 in human tissue [28]. Most authors used morphological Gb3 visualization in semithin or ultrathin sections. No study applied a morphometric method for quantification. One crucial advantage of visualizing Gb3 in skin by immunofluorescence is that this technique is much easier to perform and more widely available. Also, equipment and assessment costs are markedly lower than that of electron microscopy. Moreover, when using immunofluorescence the entire skin biopsy specimen can be evaluated while electron microscopy allows a structurally detailed, but spatially limited look at only a very small piece of skin. Thus a sampling error is less likely. Furthermore, an immunofluorescent stain can be quantified by automatic or semiautomatic morphometry, which allows unbiased quantification of Gb3 load. On the other hand, Gb3 immunofluorescence appears to be less sensitive than electron microscopy (see below), at least with the antibodies and methods used in the present study.

Although skin is easily accessible and skin punch biopsy is minimally invasive, there is scarce information on the correlation of dermal Gb3 deposits with FD severity. Several studies investigated treatment response to ERT. Eng et al. saw Gb3 clearance in skin from the back 20 weeks after the initiation of ERT in a placebo controlled trial [29]. One further long-term and one small short-term study assessed skin Gb3 under ERT and showed partial clearance [10,30]. In a recent study using electron microscopy, Gb3 deposits were described and semiquantitatively assessed in treatment-naïve male pediatric FD patients [8]. Interestingly, Gb3 was found in superficial skin capillary endothelial cells and deep vessel endothelial cells of the majority of these young male patients, which is quantitatively similar to our findings with 53% male patients showing Gb3 deposits of >0.05% ROI in distal skin, however, with less spatial resolution as a disadvantage of the method.

Diagnostic guidelines increasingly propose the demonstration of Gb3 deposits in affected organs including skin in patients with uncertain FD or with a genetic variant of unknown significance [31]. Thus, a positive Gb3 skin finding in a patient with a genetic variant would support the FD diagnosis in these patients. In our cohort, we had seven patients with genetic variants that together with the clinical phenotype and laboratory findings led to the grouping as “non-classic”. Interestingly distal cutaneous Gb3 deposits were similar to controls in contrast to patients with classic FD (see Fig 3).

The effects of Gb3 deposits in skin of FD patients are not well understood yet. Due to the topical proximity of dermal Gb3 to skin nerve fibers, an influence on skin innervation and/or skin nerve fiber sensitization may be assumed. Here we found a higher Gb3 load in patients with SFN, however, no linear correlation was present between Gb3 load and IENFD. The relatively small number of cases per subgroup made statistical analysis difficult, thus, correlations might have turned positive if more cases would have been investigated in each subgroup. On the other hand it is not known whether cutaneous Gb3 deposits are causally related to small fiber degeneration or if Gb3 deposits in the dorsal root ganglia disturbing neuronal function might correlate with nerve fiber loss [32]. Gb3 deposits may influence sweating by either mechanical obstruction of sweat glands or by disturbance of sweat gland innervation leading to the FD phenotype of hypo- to anhidrosis.

We did not find a difference in the Gb3 load of patients on ERT compared to patients without ERT. Factors that may have led to this rather unexpected finding are the low number of subjects in each of our subgroups. Furthermore, the time span of patients on ERT was large ranging from patients who had just received their first infusions to those who were on ERT for almost a decade. Again, subgroups were too small to perform biologically meaningful statistical analyses. To clarify the effect of ERT on skin Gb3 systematic studies in a large patient cohort stratified for treatment duration are needed.

One limitation of our study is that although the entire patient cohort was large, stratification for gender and different aspects of disease severity led to small subgroups, which in some instances hindered reaching the necessary statistical power. The strength of our study is that we have investigated a clinically very well characterized single-center Fabry patient cohort comparing our results not only with appropriate patient subgroups but also with healthy controls. Also, we introduce Gb3 immunofluorescence as an easy-to-perform and reliable new application that is less sensitive than electronmicroscopy, however, specifically indicates Gb3 deposits and indicates high disease severity. The determination of skin Gb3 load is therefore a potential new tool for diagnostics and treatment stratification in FD patients.

## Supporting Information

S1 Table Individual patient data. (DOC)

S2 Table Individual patient data on kidney function. (DOC)

## Abbreviations

ADS: Allgemeine Depressionsskala
ERT: enzyme replacement therapy
F: female
M: male
NPSI: Neuropathic Pain Symptom Inventory
SFN: small fiber neuropathy

## References

[1] Schiffmann R. Fabry disease. Pharmacol Ther. 2009;122: 65–77. 10.1016/j.pharmthera.2009.01.003 19318041

[2] Lao LM, Kumakiri M, Mima H, Kuwahara H, Ishida H, Ishiguro K, et al The ultrastructural characteristics of eccrine sweat glands in a Fabry disease patient with hypohidrosis. J Dermatol Sci. 1998;18: 109–117. 9833977 10.1016/s0923-1811(98)00032-2

[3] Palungwachira P, Yaguchi H. The ultrastructural study in a case of Fabry disease. J Med Assoc Thai. 2002;85: 842–849. 12296419

[4] Kanekura T, Fukushige T, Kanda A, Tsuyama S, Murata F, Sakuraba H, et al Immunoelectron-microscopic detection of globotriaosylceramide accumulated in the skin of patients with Fabry disease. Br J Dermatol. 2005;153: 544–548. 10.1111/j.1365-2133.2005.06732.x 16120140

[5] van Mullem PJ. Ultrastructure of lipid bodies and lysosomes in the skin in Fabry's disease (angiokeratoma corporis diffusum). Arch Belg Dermatol Syphiligr. 1972;28: 41–49. 4348059

[6] Askari H, Kaneski CR, Semino-Mora C, Desai P, Ang A, Kleiner DE, et al Cellular and tissue localization of globotriaosylceramide in Fabry disease. Virchows Arch. 2007;451: 823–834. 10.1007/s00428-007-0468-6 17674039

[7] Lakoma J, Donadio V, Liguori R, Caprini M. Characterization of Human Dermal Fibroblasts in Fabry Disease. J Cell Physiol. 2016;231: 192–203. 10.1002/jcp.25072 26058984

[8] Wijburg FA, Benichou B, Bichet DG, Clarke LA, Dostalova G, Fainboim A, et al Characterization of early disease status in treatment-naive male paediatric patients with fabry disease enrolled in a randomized clinical trial. PLoS ONE. 2015;10: e0124987 10.1371/journal.pone.0124987 25955246 PMC4425695

[9] Navarro C, Teijeira S, Dominguez C, Fernandez JM, Rivas E, Fachal C, et al Fabry disease: an ultrastructural comparative study of skin in hemizygous and heterozygous patients. Acta Neuropathol (Berl). 2006;111: 178–185. 16463201 10.1007/s00401-005-0026-8

[10] Thurberg BL, Randolph Byers H, Granter SR, Phelps RG, Gordon RE, O'Callaghan M. Monitoring the 3-year efficacy of enzyme replacement therapy in fabry disease by repeated skin biopsies. J Invest Dermatol. 2004;122: 900–908. 10.1111/j.0022-202X.2004.22425.x 15102080

[11] Tondel C, Bostad L, Larsen KK, Hirth A, Vikse BE, Houge G, et al Agalsidase benefits renal histology in young patients with Fabry disease. J Am Soc Nephrol. 2013;24: 137–148. 10.1681/ASN.2012030316 23274955 PMC3537211

[12] Valbuena C, Leitao D, Carneiro F, Oliveira JP. Immunohistochemical diagnosis of Fabry nephropathy and localisation of globotriaosylceramide deposits in paraffin-embedded kidney tissue sections. Virchows Arch. 2012;460: 211–221. 10.1007/s00428-011-1182-y 22205110

[13] Üçeyler N, He L, Schönfeld D, Kahn AK, Reiners K, Hilz MJ, et al Small fibers in Fabry disease: baseline and follow-up data under enzyme replacement therapy. J Peripher Nerv Syst. 2011;16: 304–314. 10.1111/j.1529-8027.2011.00365.x 22176145

[14] Üçeyler N, He L, Kahn AK, Breunig F, Mullges W, Sommer C. Cerebral blood flow in patients with Fabry disease as measured by Doppler sonography is not different from that in healthy individuals and is unaffected by treatment. J Ultrasound Med. 2012;31: 463–468. 22368137 10.7863/jum.2012.31.3.463

[15] Üçeyler N, Homola GA, Guerrero González H, Kramer D, Wanner C, Weidemann F, et al Increased arterial diameters in the posterior cerebral circulation in men with fabry disease. PLoS One. 2014;9: e87054 10.1371/journal.pone.0087054 24475221 PMC3903616

[16] Bouhassira D, Attal N, Fermanian J, Alchaar H, Gautron M, Masquelier E, et al Development and validation of the Neuropathic Pain Symptom Inventory. Pain. 2004;108: 248–257. 10.1016/j.pain.2003.12.024 15030944

[17] Sommer C, Richter H, Rogausch JP, Frettloh J, Lungenhausen M, Maier C. A modified score to identify and discriminate neuropathic pain: a study on the German version of the neuropathic pain symptom inventory (NPSI). BMC Neurol. 2011;11: 104 10.1186/1471-2377-11-104 21861889 PMC3180265

[18] Devigili G, Tugnoli V, Penza P, Camozzi F, Lombardi R, Melli G, et al The diagnostic criteria for small fibre neuropathy: from symptoms to neuropathology. Brain. 2008;131: 1912–1925. 10.1093/brain/awn093 18524793 PMC2442424

[19] Rolke R, Baron R, Maier C, Tölle TR, Treede RD, Beyer A, et al Quantitative sensory testing in the German Research Network on Neuropathic Pain (DFNS): standardized protocol and reference values. Pain. 2006;123: 231–243. 10.1016/j.pain.2006.01.041 16697110

[20] Schiffmann R, Warnock DG, Banikazemi M, Bultas J, Linthorst GE, Packman S, et al Fabry disease: progression of nephropathy, and prevalence of cardiac and cerebrovascular events before enzyme replacement therapy. Nephrol Dial Transplant. 2009;24: 2102–2111. 10.1093/ndt/gfp031 19218538 PMC2698092

[21] Tahir H, Jackson LL, Warnock DG. Antiproteinuric Therapy and Fabry Nephropathy: Sustained Reduction of Proteinuria in Patients Receiving Enzyme Replacement Therapy with Agalsidase-beta. J Am Soc Nephrol. 2007;18: 2609–2617. 10.1681/ASN.2006121400 17656478

[22] Vedder AC, Linthorst GE, Houge G, Groener JE, Ormel EE, Bouma BJ, et al Treatment of Fabry disease: outcome of a comparative trial with agalsidase alfa or beta at a dose of 0.2 mg/kg. PLoS ONE. 2007;2: e598 10.1371/journal.pone.0000598 17622343 PMC1913555

[23] McCarthy BG, Hsieh ST, Stocks A, Hauer P, Macko C, Cornblath DR, et al Cutaneous innervation in sensory neuropathies: evaluation by skin biopsy. Neurology. 1995;45: 1848–1855. 7477980 10.1212/wnl.45.10.1848

[24] Üçeyler N, Kafke W, Riediger N, He L, Necula G, Toyka KV, et al Elevated proinflammatory cytokine expression in affected skin in small fiber neuropathy. Neurology. 2010;74: 1806–1813. 10.1212/WNL.0b013e3181e0f7b3 20513817

[25] Lauria G, Cornblath DR, Johansson O, McArthur JC, Mellgren SI, Nolano M, et al EFNS guidelines on the use of skin biopsy in the diagnosis of peripheral neuropathy. Eur J Neurol. 2005;12: 747–758. 10.1111/j.1468-1331.2005.01260.x 16190912

[26] Tetaud C, Falguieres T, Carlier K, Lecluse Y, Garibal J, Coulaud D, et al Two distinct Gb3/CD77 signaling pathways leading to apoptosis are triggered by anti-Gb3/CD77 mAb and verotoxin-1. J Biol Chem. 2003;278: 45200–45208. 10.1074/jbc.M303868200 12944404

[27] van der Tol L, Sminia ML, Hollak CE, Biegstraaten M. Cornea verticillata supports a diagnosis of Fabry disease in non-classical phenotypes: results from the Dutch cohort and a systematic review. Br J Ophthalmol. 2016;100: 3–8. 10.1136/bjophthalmol-2014-306433 25677671

[28] Rozenfeld PA, Croxatto O, Ebner R, Fossati CA. Immunofluorescence detection of globotriaosylceramide deposits in conjunctival biopsies of Fabry disease patients. Clin Experiment Ophthalmol. 2006;34: 689–694. 10.1111/j.1442-9071.2006.01318.x 16970764

[29] Eng CM, Banikazemi M, Gordon RE, Goldman M, Phelps R, Kim L, et al A phase 1/2 clinical trial of enzyme replacement in fabry disease: pharmacokinetic, substrate clearance, and safety studies. Am J Hum Genet. 2001;68: 711–722. 10.1086/318809 11179018 PMC1274483

[30] Wraith JE, Tylki-Szymanska A, Guffon N, Lien YH, Tsimaratos M, Vellodi A, et al Safety and efficacy of enzyme replacement therapy with agalsidase beta: an international, open-label study in pediatric patients with Fabry disease. J Pediatr. 2008;152: 563–570, 570 e561 10.1016/j.jpeds.2007.09.007 18346516

[31] van der Tol L, Svarstad E, Ortiz A, Tondel C, Oliveira JP, Vogt L, et al Chronic kidney disease and an uncertain diagnosis of Fabry disease: approach to a correct diagnosis. Mol Genet Metab. 2015;114: 242–247. 10.1016/j.ymgme.2014.08.007 25187469

[32] Kaye EM, Kolodny EH, Logigian EL, Ullman MD. Nervous system involvement in Fabry's disease: clinicopathological and biochemical correlation. Ann Neurol. 1988;23: 505–509. 10.1002/ana.410230513 3133979

