# Supplementary material for: Skin Globotriaosylceramide 3 Load Is Increased in Men with Advanced Fabry Disease
Source: PLoS One. 2016 Nov 16;11(11):e0166484. doi: 10.1371/journal.pone.0166484 (PMC5112934; doi:10.1371/journal.pone.0166484)
Supplement: S1 Table — (DOC) [file pone.0166484.s001.doc]

**Supplementary Table 1:** Individual patient data.

| **Pati-ent-ID** | **Gender** | **Age** | **Genotype** | **Alpha-galactosidase activity (nmol/min/mg protein) (nk: not known)** | **Cornea**  **Verticillata (+: positive; -: negative; nk: not known)** | **Angioceratoma (+: positive; -: negative; nk: not known)** | **Symptom characterisitc of Fabry disease (pain, heart, kidney) (+: positive; -: negative)** | **Lyso Gb3 level (ng/ml) (nk: not known)** | **Family history (+: positive; -: negative; nk: not known)** | **Classic/**  **Non-Classic** |
| --- | --- | --- | --- | --- | --- | --- | --- | --- | --- | --- |
| 1 | m | 39 | W399X Exon 7, point mutation c. 1196G>A // W399X | 0.02 | nk | - | + | 107.0 | + | Classic |
| 2 | m | 39 | Exon 6, deletion c.972del G | 0.01 | nk | + | + | 32.3 | + | Classic |
| 3 | m | 63 | Exon 6, c. 931 C>G, mutation L311V + S236G of MYBPC3 | 0.06 | nk | - | + | 53.6 | nk | Classic |
| 4 | m | 53 | Exon 4, point mutation c. 611 G>A // W204X | 0.03 | nk | + | + | 102.0 | + | Classic |
| 5 | m | 60 | Exon 2, point mutation c.335 G>A // R112H | 0.02 | nk | - | + | 2.1 | - | Classic |
| 6 | m | 71 | N215S Exon 5, point mutation transition c.644 A>G // N215S | 0.05 | nk | - | + | 16.3 | nk | Classic |
| 7 | m | 31 | Exon 7, point mutation // W349X | 0.02 | + | + | + | 66.2 | + | Classic |
| 8 | m | 49 | Exon 3, point mutation c.494A>T // D165V | 0.01 | + | + | + | Nk | nk | Classic |
| 9 | m | 41 | Exon 7, mutation c.1208 del | 0.02 | + | + | + | 15.0 | + | Classic |
| 10 | m | 54 | Intron 6, transition IVS6-10G>A + polymorphisms IVS6-22 C>T | 0.04 | + | + | + | 28.1 | + | Classic (Fabry cardiomyopathy, dialysis, cerebral stroke, neuropathic pain, positive family history) |
| 11 | m | 47 | Exon 1, point mutation c.162del T | 0.01 | nk | + | + | 30.6 | + | Classic |
| 12 | m | 58 | Exon 6, transition c.845C>T, point mutation T282I + several polymorphisms | 0.03 | - | - | + | 25.9 | + | Classic |
| 13 | m | 38 | Exon 3, point mutation IVS3+1 | 0.01 | + | - | + | nk | + | Classic |
| 14 | m | 38 | IVS0-10C>T, IVS4-16A>G, IVS6-22 C>T | 0.04 | - | - | + | 0.0 | nk | Non-classic |
| 15 | m | 18 | Exon 7, point mutation // W349X | 0.01 | nk | nk | + | 312.0 | + | Classic |
| 16 | m | 26 | Exon 3, point mutation transition c.515 G>A // C172Y | 0.04 | nk | + | + | 150.0 | + | Classic |
| 17 | m | 29 | Exon 5, c.784T>C p.W262R | 0.05 | nk | + | + | 209.0 | + | Classic |
| 18 | m | 65 | Exon 5, point mutation transition c.644 A>G // N215S | 0.01 | nk | nk | + | 8.2 | + | Classic |
| 19 | m | 25 | Exon 6, point mutation c.934 C>T //Q312X | 0.06 | - | + | + | 71.7 | + | Classic |
| 20 | m | 31 | Exon 6, c.993_994 ins A (fs X 338) | 0.02 | nk | + | + | nk | + | Classic |
| 21 | m | 29 | Exon 7, mutation deletion c.1029_1030 del TC fs X30 | 0.02 | nk | - | + | 94.2 | nk | Classic |
| 22 | m | 34 | Exon 3, point mutation transition c.427 G>A // A143T | 0.02 | - | - | + | 0.6 | + | Classic |
| 23 | m | 47 | Exon 2, deletion | 0.03 | nk | + | + | 172.0 | nk | Classic |
| 24 | m | 51 | Exon 6, point mutation c.937 G>T // D313Y | 0.3 | - | - | + | 0.0 | + | Non-classic |
| 25 | m | 21 | Exon 5, mutation c.756 or 757 del A, fs 268X | 0.04 | nk | + | + | 121.0 | + | Classic |
| 26 | m | 24 | Exon 3, point mutation c.508 G>A // D170N | 0.02 | - | + | + | 72.1 | + | Classic |
| 27 | m | 51 | Exon 5, point mutation c.708 G>C //p.W236C | 0.00 | + | + | + | 95.8 | nk | Classic |
| 28 | m | 34 | Intron 6, transition IVS6-10G>A (splice –site-mutation c. 1000-10G>A) | 0.04 | - | - | + | 68.2 | + | Classic (Fabry cardiomyopathy, cerebral stroke, small fiber neuropathy) |
| 29 | m | 46 | Exon 3, point mutation transition c.404 C>T // A135V | 0.02 | + | + | + | 83.3 | + | Classic |
| 30 | m | 41 | Exon 7, point mutation c.1025 G>T // R342L | 0.03 | + | + | + | 50.5 | + | Classic |
| 31 | m | 27 | Exon 7. point mutation c. 1069 C>T  Gln357X | 0.03 | nk | + | + | 122.0 | + | Classic |
| 32 | m | 42 | Exon 3, point mutation c.408 T>A // D136E | 0.02 | - | + | + | 42.3 | + | Classic |
| 33 | m | 36 | Exon 5, point mutation c.679 C>T // R227X | 0.02 | + | + | + | 85.0 | - | Classic |
| 34 | m | 56 | Exon 3, point mutation c.386 T>C // L129P | 0.01 | + | + | + | 21.2 | + | Classic |
| 35 | m | 51 | Exon 1. 42del TGCGCTT | 0.05 | + | + | + | 42.8 | + | Classic |
| 36 | m | 40 | Exon 3, point mutation IVS3+1 Intron 3, IVS3+1 G>A | 0.00 | - | + | + | nk | + | Classic |
| 37 | m | 41 | Exon 7, mutation c.1208 del | 0.03 | + | + | + | 66.2 | + | Classic |
| 38 | m | 27 | Exon 3, point mutation c.386 T>C // L129P | 0.03 | nk | + | + | 74.1 | + | Classic |
| 39 | w | 69 | Exon 6, point mutation c.973 G>A // G325S | 0.2 | - | - | + | 4.5 | + | Classic |
| 40 | w | 48 | Exon 6, c.993_994 ins A (fs X 338) | 0.3 | - | - | + | 3.5 | + | Classic |
| 41 | w | 39 | Exon 4, point mutation c.612 G>T  // W204C + polymorphisms | 0.1 | - | - | + | 8.9 | + | Classic |
| 42 | w | 47 | -10C>T homoz.IVS4-16 A>G homoz. IVS6-22 C>T homoz Exon 7. c.1196G>C (het) p.W399S + Intron3, IVS2-81_77 homo. -10C>T homoz.IVS4-16 A>G homocygote IVS6-22 C>T homocygote | 0.3 | nk | - | + | 0.7 | nk | Classic (positive kidney biopsy) |
| 43 | w | 69 | Exon 5, mutation  c.756 or 757 del A. fs 268X | 0.2 | + | - | + | 18.8 | + | Classic |
| 44 | w | 39 | Exon 5, point mutation c.708G>C // W236C | 0.3 | - | - | + | 8.8 | + | Classic |
| 45 | w | 32 | Exon 3, point mutation transition c.515 G>A // C172Y | 0.3 | nk | - | - | 5.6 | + | Classic |
| 46 | w | 35 | Intron 3, IVS2-81 -77 + IVS0-10C>T. IVS4-16A>G. IVS6-22 C>T | 0.3 | - | - | - | 0.70 | + | Non-classic |
| 47 | w | 48 | Exon 1, point mutation c.137 A>G // H46R | 0.4 | + | + | + | 15.9 | + | Classic |
| 48 | w | 22 | Exon 7, deletion c.1221 del A | 0.3 | + | - | + | 6.9 | + | Classic |
| 49 | w | 31 | Exon 7, deletion c.1221 del A | 0.2 | - | + | + | 23.4 | + | Classic |
| 50 | w | 21 | Exon 5, point mutation c.756 or 757 del A. fs 268X | 0.2 | - | - | - | nk | + | Classic |
| 51 | w | 34 | Exon 6, c.993_994 ins A (fs X 338) | 0.2 | + | - | + | 8.4 | + | Classic |
| 52 | w | 26 | Exon 4, point mutation c. 611 G>A // W204X | 0.3 | nk | - | + | 6.4 | + | Classic |
| 53 | w | 57 | Exon 3, point mutation transition c.515 G>A // C172Y | 0.4 | - | + | + | 20.1 | + | Classic |
| 54 | w | 37 | Exon 3, point mutation c.408 T>A // D136E | 0.2 | + | - | + | 2.8 | + | Classic |
| 55 | w | 47 | Exon 6, point mutation, transition c.838 C>T // Q280X | 0.01 | + | - | + | Nk | + | Classic |
| 56 | w | 31 | Exon 3, point mutation transition c.515 G>A // C172Y | 0.2 | nk | + | + | 8.1 | + | Classic |
| 57 | w | 30 | Intron 6, transition IVS6-10G>A (splice -site-mutation c. 1000-10G>A) | 0.4 | nk | - | + | 6.0 | + | Classic |
| 58 | w | 37 | Exon 3, point mutation c.408 T>A // D136E | 0.2 | nk | - | + | 0.7 | + | Classic |
| 59 | w | 21 | Exon 6, point mutation c.937 G>T // D313Y | nk | - | - | - | 0.2 | + | Non-classic |
| 60 | w | 24 | Exon 5, point mutation c.671 A>G. // N224S | 0.3 | nk | - | - | 3.9 | + | Classic |
| 61 | w | 55 | Exon 7, point mutation, deletion c.1221 del A | 0.1 | nk | - | + | 24.3 | + | Classic |
| 62 | w | 40 | Intron 3, IVS2-81-77 + IVS0-10C>T. IVS4-16A>G. IVS6-22 C>T | 0.2 | - | - | - | 0.70 | + | Non-classic |
| 63 | w | 39 | Exon 3, point mutation tTransition c.427 G>A // A143T | 0.4 | - | - | + | 0.4 | + | Classic |
| 64 | w | 48 | Exon 3, point mutation transition c.404 C>T // A135V | 0.3 | + | - | + | 7.6 | + | Classic |
| 65 | w | 54 | IVS0-10C>T. IVS4-16A>G. IVS6-22 C>T | 0.4 | - | - | + | 0.8 | + | Classic |
| 66 | w | 62 | Exon 5, point mutation transition c.644 A>G // N215S | 0.4 | - | - | + | 1.4 | + | Classic |
| 67 | w | 59 | Exon 7, deletion c.1221 del A | 0.3 | + | - | + | 9.6 | + | Classic |
| 68 | w | 65 | Exon 3, point mutation transition c.427 G>A // A143T | 0.4 | - | - | - | 0.2 | + | Classic |
| 69 | w | 58 | Exon 6, c.993_994 ins A (fs X 338) | 0.1 | nk | - | + | 5.7 | + | Classic |
| 70 | w | 21 | Exon 7, point mutation c. 1069 C>T //  Gln357X | 0.1 | nk | - | + | 12.3 | + | Classic |
| 71 | w | 67 | Exon 7, mutation c.1208 del | 0.4 | - | - | + | 0.5 | + | Classic |
| 72 | w | 55 | Intron 6, transition IVS6-10G>A (splice -site-mutation c. 1000-10G>A) | 0.3 | nk | - | + | 7.2 | + | Classic |
| 73 | w | 21 | Exon 3, point transition c.404 C>T // A135V | 0.1 | + | - | + | 12.2 | + | Classic |
| 74 | w | 46 | Exon 6, point mutation c.973 G>A. // G325S +polymorphisms | 0.5 | nk | - | + | 8.3 | + | Classic |
| 75 | w | 49 | Exon 3, point mutation c.416 A>G // N139S | 0.2 | nk | - | - | 2.5 | + | Classic |
| 76 | w | 40 | c.378A>G (p.S126G) + polymorphisms | 0.4 | nk | - | - | nk | + | Non-classic |
| 77 | w | 37 | Exon 3, point mutation transition c.427 G>A // A143T | 0.3 | - | - | - | 0.3 | + | Classic |
| 78 | w | 34 | Exon 4, point mutation c.559 A>G // M187V | 0.4 | - | - | - | nk | + | Classic |
| 79 | w | 48 | Exon 7, point mutation deletion 354fsdel 15bp | 0.02 | + | - | + | 10.1 | nk | Classic |
| 80 | w | 20 | Exon 6, point mutation c.937G>T // D313Y | 0.4 | nk | - | - | 0.8 | + | Non-classic |
| 81 | w | 43 | Exon 3, point mutation c.386 T>C // L129P | 0.2 | - | + | + | 9.5 | + | Classic |
| 82 | w | 45 | Exon 6, IVS5-3_2del CA | 0.1 | + | - | + | 18.2 | + | Classic |
| 83 | w | 65 | Exon 3, point mutation c.408 T>A // D136E | 0.04 | - | - |  | 10.3 | + | Classic |
| 84 | w | 41 | Exon 5, point mutation c.679 C>T // R227X | 0.2 | + | + | + | 8.0 | + | Classic |
