# Supplementary material for: Skin Globotriaosylceramide 3 Load Is Increased in Men with Advanced Fabry Disease
Source: PLoS One. 2016 Nov 16;11(11):e0166484. doi: 10.1371/journal.pone.0166484 (PMC5112934; doi:10.1371/journal.pone.0166484)
Supplement: S2 Table — (DOC) [file pone.0166484.s002.doc]

**Supplementary Table 2:** Individual patient data on kidney function.

| **ID#** | **Gender** | **Age [years]** | **Glomerular filtration rate [ml/min]** | **Albuminuria [mg/dl]** | **Proteinuria [mg/dl]** | **CKD stadium** |
| --- | --- | --- | --- | --- | --- | --- |
| 1 | m | 39 | 56 | 2442 | 2809 | 3b |
| 2 | m | 39 | Dialysis | Dialysis | Dialysis | 5 |
| 3 | m | 63 | 59 | 148 | 260 | 3a |
| 4 | m | 53 | 25 | 1142 | 1169 | 4 |
| 5 | m | 60 | 60 | 21 | 107 | 2 |
| 6 | m | 71 | 53 | 1463 | 1526 | 3a |
| 7 | m | 31 | 85 | 2489 | 3059 | 2 |
| 8 | m | 49 | 7 | No data | 3402 | 5 |
| 9 | m | 41 | 20 | 2310 | 6576 | 4 |
| 10 | m | 54 | Dialysis | Dialysis | Dialysis | 5 |
| 11 | m | 47 | 41 | No data | 2300 | 3b |
| 12 | m | 58 | 37 | 779 | 1212 | 3b |
| 13 | m | 38 | 160 | <12 | 83 | 1 |
| 14 | m | 38 | 115 | <12 | 100 | 1 |
| 15 | m | 18 | 69 | 34 | 130 | 2 |
| 16 | m | 26 | 109 | 46 | 119 | 1 |
| 17 | m | 29 | 130 | 105 | 244 | 1 |
| 18 | m | 65 | 103 | <12 | 56 | 1 |
| 19 | m | 25 | 110 | <12 | 88 | 1 |
| 20 | m | 31 | 105 | 88 | 148 | 1 |
| 21 | m | 29 | 85 | 1458 | 1600 | 2 |
| 22 | m | 34 | 118 | <12 | <40 | 1 |
| 23 | m | 47 | 59 | 1537 | 1601 | 3a |
| 24 | m | 51 | 108 | 12 | 104 | 1 |
| 25 | m | 21 | 132 | 39 | 48 | 1 |
| 26 | m | 24 | 150 | 72 | 86 | 1 |
| 27 | m | 51 | 76 | 1636 | 2142 | 2 |
| 28 | m | 34 | 91 | 37 | 140 | 1 |
| 29 | m | 46 | 112 | 530 | 583 | 1 |
| 30 | m | 41 | 92 | 780 | 1550 | 1 |
| 31 | m | 27 | 96 | <12 | 80 | 1 |
| 32 | m | 42 | Dialysis | Dialysis | Dialysis | 5 |
| 33 | m | 36 | 91 | <12 | <30 | 1 |
| 34 | m | 56 | 69 | No data | 1293 | 2 |
| 35 | m | 51 | 95 | 465 | 550 | 1 |
| 36 | m | 40 | 225 | <12 | <30 | 1 |
| 37 | m | 41 | 133 | 1146 | 1914 | 1 |
| 38 | m | 27 | 145 | <12 | <40 | 1 |
| 39 | w | 69 | 58 | 31 | 80 | 3a |
| 40 | w | 48 | 82 | <12 | 127 | 2 |
| 41 | w | 39 | 41 | 723 | 1217 | 3b |
| 42 | w | 47 | 52 | 554 | 882 | 3a |
| 43 | w | 69 | 40 | 1888 | 2365 | 3b |
| 44 | w | 39 | 102 | 76 | 142 | 1 |
| 45 | w | 32 | 88 | <12 | <40 | 2 |
| 46 | w | 35 | 76 | <12 | <40 | 2 |
| 47 | w | 48 | 82 | 99 | 175 | 2 |
| 48 | w | 22 | 112 | <12 | 37 | 1 |
| 49 | w | 31 | 104 | <12 | 81 | 1 |
| 50 | w | 21 | 96 | 54 | 173 | 1 |
| 51 | w | 34 | 102 | <12 | <40 | 1 |
| 52 | w | 26 | 92 | <12 | 54 | 1 |
| 53 | w | 57 | 92 | 9.8 | 43 | 1 |
| 54 | w | 37 | 102 | 9 | <30 | 1 |
| 55 | w | 47 | 221 | <12 | <30 | 1 |
| 56 | w | 31 | 89 | 56 | 113 | 2 |
| 57 | w | 30 | 78 | 9 | 46 | 2 |
| 58 | w | 37 | 101 | <12 | 88 | 1 |
| 59 | w | 21 | 112 | 39 | 99 | 1 |
| 60 | w | 24 | 131 | 17 | 73 | 1 |
| 61 | w | 55 | 61 | 90 | 157 | 2 |
| 62 | w | 40 | 74 | No data | No data | 2 |
| 63 | w | 39 | 74 | <12 | 165 | 2 |
| 64 | w | 48 | 98 | 14 | 35 | 1 |
| 65 | w | 54 | 80 | <12 | <40 | 2 |
| 66 | w | 62 | 77 | <12 | <40 | 2 |
| 67 | w | 59 | 68 | 316 | 448 | 2 |
| 68 | w | 65 | 89 | <12 | <40 | 2 |
| 69 | w | 58 | 61 | <12 | 57 | 2 |
| 70 | w | 21 | 96 | <12 | 63 | 1 |
| 71 | w | 67 | 128 | <12 | <30 | 1 |
| 72 | w | 55 | 69 | 81.6 | 140 | 2 |
| 73 | w | 21 | 96 | <12 | 55 | 1 |
| 74 | w | 46 | 96 | 12 | 40 | 1 |
| 75 | w | 49 | 95 | 56.4 | 104 | 1 |
| 76 | w | 40 | 74 | No data | No data | No data |
| 77 | w | 37 | 121 | <12 | 182 | 1 |
| 78 | w | 34 | 81 | No data | No data | No data |
| 79 | w | 48 | 97 | <12 | <30 | 1 |
| 80 | w | 20 | 98 | <12 | <40 | 1 |
| 81 | w | 43 | 117 | 15 | 74 | 1 |
| 82 | w | 45 | 72 | 20 | 106 | 2 |
| 83 | w | 65 | 98 | 919 | 1341 | 1 |
| 84 | w | 41 | 87 | <12 | <30 | 2 |
